# Ethyl 4-(4-bromo­phen­yl)-6-*r*-phenyl-2-oxocyclo­hex-3-ene-1-*t*-carboxyl­ate

**DOI:** 10.1107/S1600536810025353

**Published:** 2010-07-03

**Authors:** N. Anuradha, A. Thiruvalluvar, C. Yuvaraj, K. Pandiarajan, R. J. Butcher

**Affiliations:** a PG Research Department of Physics, Rajah Serfoji Government College (Autonomous), Thanjavur 613 005, Tamil Nadu, India; b Department of Chemistry, Annamalai University, Annamalai Nagar 608 002, Tamil Nadu, India; c Department of Chemistry, Howard University, 525 College Street NW, Washington, DC 20059, USA

## Abstract

In the title compound, C_21_H_19_BrO_3_, the cyclo­hexene ring adopts an envelope conformation, with all substituents equatorial. The plane through its five coplanar atoms makes dihedral angles of 28.88 (10) and 71.94 (10)° with the bromo­benzene and phenyl rings, respectively. The dihedral angle between the latter two rings is 51.49 (15)°. Inter­molecular C—H⋯O hydrogen bonds are found in the crystal structure; a C—H⋯π inter­action is also present.

## Related literature

For the synthesis of cyclo­hexenone derivatives, see: Chong *et al.* (1997 ▶); Inokuchi *et al.* (2001 ▶). For their applications and for related structures, see: Anuradha *et al.* (2009 ▶); Fun *et al.* (2010 ▶).
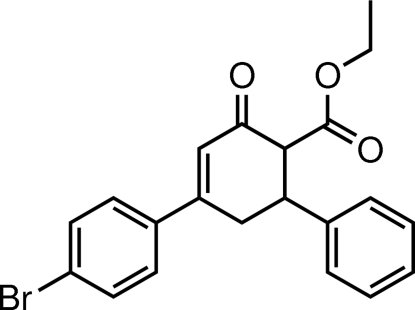


## Experimental

### 

#### Crystal data

C_21_H_19_BrO_3_
*M* *_r_* = 399.26
Monoclinic, 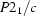

*a* = 11.0138 (2) Å
*b* = 13.8197 (4) Å
*c* = 12.1477 (3) Å
β = 95.180 (2)°
*V* = 1841.42 (8) Å^3^
*Z* = 4
Cu *K*α radiation
μ = 3.17 mm^−1^
*T* = 295 K
0.44 × 0.36 × 0.12 mm

#### Data collection

Oxford Diffraction Xcalibur Ruby Gemini diffractometer
Absorption correction: multi-scan (*CrysAlis PRO*; Oxford Diffraction, 2010 ▶) *T*_min_ = 0.444, *T*_max_ = 1.000
8385 measured reflections
3851 independent reflections
3210 reflections with *I* > 2σ(*I*)
*R*_int_ = 0.021

#### Refinement

*R*[*F* ^2^ > 2σ(*F* ^2^)] = 0.050
*wR*(*F* ^2^) = 0.150
*S* = 1.08
3851 reflections
227 parameters
H-atom parameters constrained
Δρ_max_ = 0.32 e Å^−3^
Δρ_min_ = −0.61 e Å^−3^

### 

Data collection: *CrysAlis PRO* (Oxford Diffraction, 2010 ▶); cell refinement: *CrysAlis PRO*; data reduction: *CrysAlis PRO*; program(s) used to solve structure: *SIR2004* (Burla *et al.*, 2005 ▶); program(s) used to refine structure: *SHELXL97* (Sheldrick, 2008 ▶); molecular graphics: *ORTEP-3* (Farrugia, 1997 ▶); software used to prepare material for publication: *PLATON* (Spek, 2009 ▶).

## Supplementary Material

Crystal structure: contains datablocks global, I. DOI: 10.1107/S1600536810025353/tk2685sup1.cif

Structure factors: contains datablocks I. DOI: 10.1107/S1600536810025353/tk2685Isup2.hkl

Additional supplementary materials:  crystallographic information; 3D view; checkCIF report

## Figures and Tables

**Table 1:** Hydrogen-bond geometry (Å, °) *Cg* is the centroid of the C41–C46 ring.

*D*—H⋯*A*	*D*—H	H⋯*A*	*D*⋯*A*	*D*—H⋯*A*
C42—H42⋯O2^i^	0.93	2.58	3.276 (3)	132
C45—H45⋯O11^ii^	0.93	2.54	3.288 (4)	138
C1—H1⋯*Cg*^iii^	0.98	2.77	3.648 (2)	150

Symmetry codes: (i) 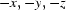; (ii) 
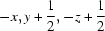
; (iii) 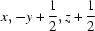.

**Fig. 1.**
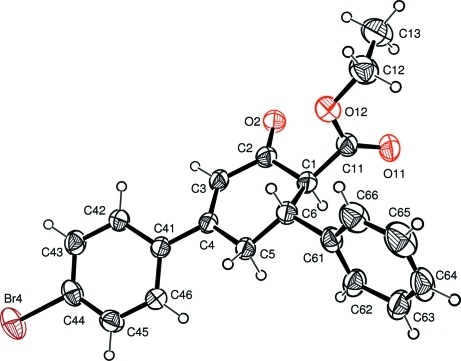
The molecular structure of the title compound, showing the atom-numbering scheme and displacement ellipsoids drawn at the 30% probability level. H atoms are shown as small spheres of arbitrary radius.

**Fig. 2.**
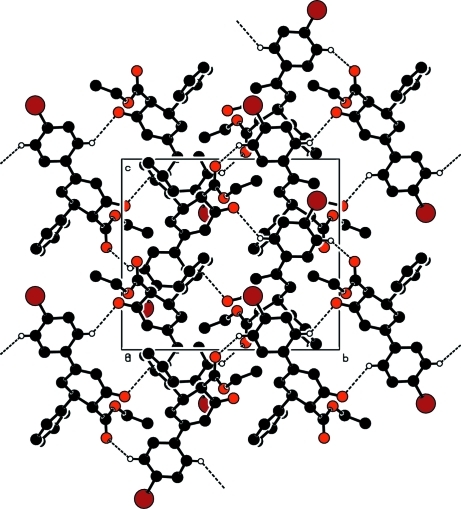
The packing of the title compound, viewed down the a axis. Dashed lines indicate hydrogen bonds. H atoms not involved in hydrogen bonding have been omitted.

## supplementary crystallographic information

### Comment

Chong *et al.* (1997) have reported highly efficient synthesis of methyl-substituted conjugate cyclohexenones. Inokuchi *et al.* (2001) have reported selective synthesis of *cis* 4,5-dimethyl-2-cyclohexenone derivatives. Anuradha *et al.* (2009) have reported a crystal structure of ethyl 6- r-(2-chlorophenyl)-2-oxo-4-phenylcyclohex-3-ene-1- t-carboxylate. Fun *et al.* (2010) have reported a crystal structure of methyl 4,6-bis(4-fluorophenyl)-2-oxocyclohex-3-ene-1-carboxylate. In the above two structures the cyclohexene rings adopt envelope conformations.

The present X-ray diffraction study was undertaken to determine how the conformation of the system is affected by the substitution of a ethoxycarbonyl group at position 1, a bromophenyl group at position 4 and a phenyl group at position 6 of the cyclohexenone ring.

In the title compound, C_21_H_19_BrO_3_, (Fig. 1), the cyclohexene ring adopts an envelope conformation with all substituents equatorial. The plane through the five coplanar atoms C1/C2/C3/C4/C5 makes dihedral angles of 28.88 (10) and 71.94 (10)° with the bromophenyl and benzene rings, respectively. The dihedral angle between the benzene and bromophenyl rings is 51.49 (15)°. C42—H42···O2(-*x*,-*y*,-*z*) and C45—H45···O11(-*x*, 1/2 + *y*, 1/2 - *z*) intermolecular hydrogen bonds are found in the crystal structure. Further, a C1—H1···π(*x*, 1/2 - *y*, 1/2 + *z*) interaction involving the benzene (C41—C46) ring is also found (Fig. 2, Table 1).

### Experimental

A mixture of benzylidene *p*-bromoacetophenone (3.03 g, 0.0125 mol), ethyl acetoacetate (2 ml, 0.0125 mol) and sodium ethoxide (1 g, 0.0125 mol) in absolute alcohol (50 ml) was refluxed for 14 h. After cooling, the reaction mixture was neutralized with 0.1 N HCl. It was then extracted with diethyl ether (3x20 ml). The organic layer was dried over anhydrous sodium sulfate, filtered and the solvents removed by rotary vacuum evaporation. A solid mass was obtained which was recrystallized from ethanol. Yield 2 g (55%).

### Refinement

H atoms were positioned geometrically and allowed to ride on their parent atoms, with C*sp*^2^—H = 0.93, C(methyl)—H = 0.96, C(methylene)—H = 0.97 and C(methine)—H = 0.98 Å; *U*_iso_(H) = k*U*_eq_(C), where k = 1.5 for methyl and 1.2 for all other H atoms.

### Crystal data

**Table:**

C_21_H_19_BrO_3_	*F*(000) = 816
*M**_r_* = 399.26	*D*_x_ = 1.440 Mg m^−^^3^
Monoclinic, *P*2_1_/*c*	Melting point: 359 K
Hall symbol: -P 2ybc	Cu *K*α radiation, λ = 1.54184 Å
*a* = 11.0138 (2) Å	Cell parameters from 4868 reflections
*b* = 13.8197 (4) Å	θ = 4.9–77.3°
*c* = 12.1477 (3) Å	µ = 3.17 mm^−^^1^
β = 95.180 (2)°	*T* = 295 K
*V* = 1841.42 (8) Å^3^	Prism, colourless
*Z* = 4	0.44 × 0.36 × 0.12 mm

### Data collection

**Table:**

Oxford Diffraction Xcalibur Ruby Gemini diffractometer	3851 independent reflections
Radiation source: Enhance (Cu) X-ray Source	3210 reflections with *I* > 2σ(*I*)
graphite	*R*_int_ = 0.021
Detector resolution: 10.5081 pixels mm^-1^	θ_max_ = 77.5°, θ_min_ = 4.9°
ω scans	*h* = −13→13
Absorption correction: multi-scan (*CrysAlis PRO*; Oxford Diffraction, 2010)	*k* = −17→12
*T*_min_ = 0.444, *T*_max_ = 1.000	*l* = −14→15
8385 measured reflections	

### Refinement

**Table:**

Refinement on *F*^2^	Primary atom site location: structure-invariant direct methods
Least-squares matrix: full	Secondary atom site location: difference Fourier map
*R*[*F*^2^ > 2σ(*F*^2^)] = 0.050	Hydrogen site location: inferred from neighbouring sites
*wR*(*F*^2^) = 0.150	H-atom parameters constrained
*S* = 1.08	*w* = 1/[σ^2^(*F*_o_^2^) + (0.0892*P*)^2^ + 0.401*P*] where *P* = (*F*_o_^2^ + 2*F*_c_^2^)/3
3851 reflections	(Δ/σ)_max_ = 0.001
227 parameters	Δρ_max_ = 0.32 e Å^−^^3^
0 restraints	Δρ_min_ = −0.61 e Å^−^^3^

### Special details

**Table:**

Geometry. Bond distances, angles *etc*. have been calculated using the rounded fractional coordinates. All su's are estimated from the variances of the (full) variance-covariance matrix. The cell e.s.d.'s are taken into account in the estimation of distances, angles and torsion angles
Refinement. Refinement of *F*^2^ against ALL reflections. The weighted *R*-factor *wR* and goodness of fit *S* are based on *F*^2^, conventional *R*-factors *R* are based on *F*, with *F* set to zero for negative *F*^2^. The threshold expression of *F*^2^ > 2σ(*F*^2^) is used only for calculating *R*-factors(gt) *etc*. and is not relevant to the choice of reflections for refinement. *R*-factors based on *F*^2^ are statistically about twice as large as those based on *F*, and *R*- factors based on ALL data will be even larger.

### Fractional atomic coordinates and isotropic or equivalent isotropic displacement parameters (Å^2^)

**Table:**

	*x*	*y*	*z*	*U*_iso_*/*U*_eq_	
Br4	−0.27481 (3)	0.39721 (4)	−0.28199 (3)	0.1008 (2)	
O2	0.0731 (2)	−0.01378 (13)	0.24548 (16)	0.0754 (7)	
O11	0.2865 (2)	0.06999 (16)	0.46249 (16)	0.0814 (7)	
O12	0.3423 (2)	0.03049 (16)	0.29591 (17)	0.0801 (8)	
C1	0.1736 (2)	0.13392 (17)	0.30076 (18)	0.0551 (7)	
C2	0.0916 (3)	0.07028 (17)	0.22341 (19)	0.0585 (7)	
C3	0.0292 (2)	0.11697 (16)	0.12766 (19)	0.0557 (7)	
C4	0.0424 (2)	0.21111 (16)	0.10325 (16)	0.0493 (6)	
C5	0.1310 (2)	0.27481 (16)	0.17181 (18)	0.0547 (7)	
C6	0.2304 (2)	0.21756 (17)	0.23886 (19)	0.0555 (7)	
C11	0.2729 (3)	0.07369 (18)	0.3645 (2)	0.0625 (8)	
C12	0.4481 (3)	−0.0246 (3)	0.3446 (3)	0.0906 (14)	
C13	0.4140 (4)	−0.1191 (3)	0.3858 (4)	0.1036 (16)	
C41	−0.0332 (2)	0.25631 (16)	0.01031 (17)	0.0507 (6)	
C42	−0.0759 (2)	0.20198 (18)	−0.08178 (19)	0.0563 (7)	
C43	−0.1466 (2)	0.2436 (2)	−0.1692 (2)	0.0652 (8)	
C44	−0.1767 (2)	0.3402 (2)	−0.1636 (2)	0.0660 (8)	
C45	−0.1361 (3)	0.3956 (2)	−0.0745 (2)	0.0679 (9)	
C46	−0.0639 (3)	0.35454 (18)	0.0122 (2)	0.0606 (8)	
C61	0.3109 (2)	0.27847 (18)	0.3204 (2)	0.0591 (7)	
C62	0.2639 (3)	0.3446 (2)	0.3907 (2)	0.0695 (9)	
C63	0.3395 (4)	0.3930 (2)	0.4700 (3)	0.0869 (13)	
C64	0.4603 (4)	0.3765 (3)	0.4805 (3)	0.1013 (14)	
C65	0.5093 (4)	0.3114 (3)	0.4118 (4)	0.1105 (18)	
C66	0.4355 (3)	0.2627 (3)	0.3320 (3)	0.0862 (11)	
H1	0.12304	0.16247	0.35470	0.0661*	
H3	−0.02300	0.07976	0.08038	0.0668*	
H5A	0.08663	0.31299	0.22186	0.0657*	
H5B	0.16886	0.31918	0.12331	0.0657*	
H6	0.28296	0.18886	0.18667	0.0666*	
H12A	0.50529	−0.03357	0.28925	0.1087*	
H12B	0.48887	0.01232	0.40502	0.1087*	
H13A	0.36252	−0.11051	0.44476	0.1555*	
H13B	0.48618	−0.15375	0.41285	0.1555*	
H13C	0.37100	−0.15507	0.32702	0.1555*	
H42	−0.05654	0.13658	−0.08455	0.0676*	
H43	−0.17342	0.20688	−0.23080	0.0782*	
H45	−0.15706	0.46075	−0.07220	0.0815*	
H46	−0.03540	0.39252	0.07225	0.0727*	
H62	0.18046	0.35672	0.38471	0.0834*	
H63	0.30635	0.43742	0.51638	0.1042*	
H64	0.51027	0.40899	0.53407	0.1213*	
H65	0.59279	0.29993	0.41900	0.1324*	
H66	0.46998	0.21897	0.28568	0.1035*	

### Atomic displacement parameters (Å^2^)

**Table:**

	*U*^11^	*U*^22^	*U*^33^	*U*^12^	*U*^13^	*U*^23^
Br4	0.0719 (3)	0.1319 (4)	0.0941 (3)	0.0257 (2)	−0.0167 (2)	0.0314 (2)
O2	0.1103 (15)	0.0451 (9)	0.0678 (11)	−0.0123 (9)	−0.0079 (10)	0.0056 (8)
O11	0.1096 (16)	0.0750 (12)	0.0560 (10)	−0.0024 (11)	−0.0120 (10)	0.0079 (9)
O12	0.0890 (14)	0.0775 (13)	0.0723 (12)	0.0158 (10)	−0.0011 (10)	0.0013 (10)
C1	0.0685 (14)	0.0503 (12)	0.0456 (10)	−0.0019 (10)	0.0009 (9)	0.0000 (9)
C2	0.0775 (15)	0.0471 (12)	0.0503 (11)	−0.0053 (10)	0.0027 (10)	−0.0011 (9)
C3	0.0682 (14)	0.0496 (12)	0.0475 (11)	−0.0090 (9)	−0.0043 (9)	−0.0053 (9)
C4	0.0591 (12)	0.0482 (11)	0.0402 (9)	−0.0027 (9)	0.0030 (8)	−0.0034 (8)
C5	0.0703 (14)	0.0475 (11)	0.0451 (10)	−0.0086 (10)	−0.0021 (9)	0.0012 (8)
C6	0.0626 (13)	0.0533 (12)	0.0498 (11)	−0.0047 (10)	0.0007 (9)	0.0007 (9)
C11	0.0781 (16)	0.0532 (12)	0.0545 (13)	−0.0045 (11)	−0.0037 (11)	0.0041 (10)
C12	0.078 (2)	0.084 (2)	0.106 (3)	0.0031 (16)	−0.0120 (17)	0.0005 (18)
C13	0.095 (3)	0.083 (2)	0.129 (3)	0.0085 (19)	−0.010 (2)	0.007 (2)
C41	0.0544 (11)	0.0529 (11)	0.0445 (10)	−0.0013 (9)	0.0026 (8)	−0.0026 (8)
C42	0.0595 (12)	0.0544 (12)	0.0531 (11)	−0.0012 (9)	−0.0052 (9)	−0.0029 (9)
C43	0.0588 (13)	0.0758 (16)	0.0582 (13)	−0.0011 (11)	−0.0094 (10)	−0.0044 (11)
C44	0.0493 (12)	0.0841 (18)	0.0631 (13)	0.0084 (11)	−0.0031 (10)	0.0144 (12)
C45	0.0711 (16)	0.0611 (14)	0.0717 (16)	0.0136 (11)	0.0071 (13)	0.0066 (12)
C46	0.0731 (15)	0.0542 (12)	0.0539 (12)	0.0043 (11)	0.0021 (10)	−0.0038 (10)
C61	0.0628 (13)	0.0575 (13)	0.0550 (12)	−0.0112 (10)	−0.0059 (10)	0.0093 (10)
C62	0.0751 (16)	0.0719 (16)	0.0597 (13)	−0.0180 (13)	−0.0038 (12)	−0.0048 (12)
C63	0.124 (3)	0.0730 (19)	0.0598 (15)	−0.0308 (17)	−0.0129 (16)	0.0008 (13)
C64	0.111 (3)	0.086 (2)	0.096 (2)	−0.036 (2)	−0.051 (2)	0.0200 (19)
C65	0.076 (2)	0.104 (3)	0.143 (4)	−0.020 (2)	−0.036 (2)	0.022 (3)
C66	0.0666 (17)	0.084 (2)	0.105 (2)	−0.0064 (14)	−0.0087 (16)	0.0123 (18)

### Geometric parameters (Å, °)

**Table:**

Br4—C44	1.891 (2)	C62—C63	1.387 (5)
O2—C2	1.214 (3)	C63—C64	1.345 (6)
O11—C11	1.187 (3)	C64—C65	1.371 (6)
O12—C11	1.323 (4)	C65—C66	1.382 (6)
O12—C12	1.471 (4)	C1—H1	0.9800
C1—C2	1.523 (3)	C3—H3	0.9300
C1—C6	1.542 (3)	C5—H5A	0.9700
C1—C11	1.528 (4)	C5—H5B	0.9700
C2—C3	1.448 (3)	C6—H6	0.9800
C3—C4	1.345 (3)	C12—H12A	0.9700
C4—C5	1.508 (3)	C12—H12B	0.9700
C4—C41	1.479 (3)	C13—H13A	0.9600
C5—C6	1.525 (3)	C13—H13B	0.9600
C6—C61	1.522 (3)	C13—H13C	0.9600
C12—C13	1.460 (6)	C42—H42	0.9300
C41—C42	1.393 (3)	C43—H43	0.9300
C41—C46	1.400 (3)	C45—H45	0.9300
C42—C43	1.384 (3)	C46—H46	0.9300
C43—C44	1.379 (4)	C62—H62	0.9300
C44—C45	1.368 (4)	C63—H63	0.9300
C45—C46	1.383 (4)	C64—H64	0.9300
C61—C62	1.383 (4)	C65—H65	0.9300
C61—C66	1.384 (4)	C66—H66	0.9300
			
Br4···C63^i^	3.720 (3)	C62···H1	2.9700
Br4···C3^ii^	3.622 (2)	C63···H6^vii^	2.9800
Br4···H64^iii^	3.1100	C64···H64^ix^	2.9900
Br4···H12A^iv^	3.2500	C65···H13B^x^	3.0400
O2···O12	3.035 (3)	H1···C62	2.9700
O2···C42^v^	3.276 (3)	H1···C41^vii^	2.9000
O11···C61	3.382 (3)	H1···C42^vii^	3.0300
O11···C13	3.147 (5)	H1···C46^vii^	2.9400
O11···C45^vi^	3.288 (4)	H3···C42	2.6200
O12···C66	3.386 (5)	H3···H42	2.1500
O12···O2	3.035 (3)	H5A···C46	2.9700
O2···H46^vi^	2.6300	H5A···C62	2.7400
O2···H43^v^	2.9000	H5A···H46	2.4200
O2···H42^v^	2.5800	H5A···H62	2.2300
O11···H13A	2.6500	H5A···C43^vii^	3.0900
O11···H12B	2.5200	H5B···C46	2.8300
O11···H5B^vii^	2.8800	H5B···H46	2.4900
O11···H45^vi^	2.5400	H5B···O11^ii^	2.8800
O12···H6	2.6100	H6···O12	2.6100
C2···C44^vii^	3.589 (4)	H6···C3	2.9900
C3···Br4^vii^	3.622 (2)	H6···H66	2.3300
C11···C66	3.212 (5)	H6···C63^ii^	2.9800
C13···O11	3.147 (5)	H12A···Br4^xi^	3.2500
C42···O2^v^	3.276 (3)	H12B···O11	2.5200
C44···C2^ii^	3.589 (4)	H12B···C12^x^	3.0600
C45···O11^viii^	3.288 (4)	H12B···C13^x^	3.0500
C61···O11	3.382 (3)	H12B···H12B^x^	2.3200
C63···Br4^i^	3.720 (3)	H13A···O11	2.6500
C64···C64^ix^	3.545 (6)	H13A···C11	2.8700
C66···C11	3.212 (5)	H13A···C45^vi^	3.0700
C66···O12	3.386 (5)	H13B···C65^x^	3.0400
C3···H6	2.9900	H13C···H43^v^	2.4800
C3···H42	2.6800	H42···C3	2.6800
C5···H62	2.8300	H42···H3	2.1500
C5···H46	2.6600	H42···O2^v^	2.5800
C11···H13A	2.8700	H43···O2^v^	2.9000
C12···H12B^x^	3.0600	H43···H13C^v^	2.4800
C13···H12B^x^	3.0500	H45···O11^viii^	2.5400
C41···H1^ii^	2.9000	H46···C5	2.6600
C42···H62^ii^	3.0000	H46···H5A	2.4200
C42···H1^ii^	3.0300	H46···H5B	2.4900
C42···H3	2.6200	H46···O2^viii^	2.6300
C43···H5A^ii^	3.0900	H62···C5	2.8300
C45···H13A^viii^	3.0700	H62···H5A	2.2300
C46···H1^ii^	2.9400	H62···C42^vii^	3.0000
C46···H5A	2.9700	H64···Br4^xii^	3.1100
C46···H5B	2.8300	H64···C64^ix^	2.9900
C62···H5A	2.7400	H66···H6	2.3300
			
C11—O12—C12	117.5 (2)	C2—C3—H3	118.00
C2—C1—C6	112.10 (18)	C4—C3—H3	118.00
C2—C1—C11	110.8 (2)	C4—C5—H5A	109.00
C6—C1—C11	110.61 (19)	C4—C5—H5B	109.00
O2—C2—C1	121.2 (2)	C6—C5—H5A	109.00
O2—C2—C3	121.8 (2)	C6—C5—H5B	109.00
C1—C2—C3	116.8 (2)	H5A—C5—H5B	108.00
C2—C3—C4	123.7 (2)	C1—C6—H6	108.00
C3—C4—C5	121.35 (19)	C5—C6—H6	108.00
C3—C4—C41	120.8 (2)	C61—C6—H6	108.00
C5—C4—C41	117.81 (19)	O12—C12—H12A	109.00
C4—C5—C6	112.93 (18)	O12—C12—H12B	109.00
C1—C6—C5	110.24 (18)	C13—C12—H12A	109.00
C1—C6—C61	109.59 (19)	C13—C12—H12B	109.00
C5—C6—C61	114.11 (19)	H12A—C12—H12B	108.00
O11—C11—O12	125.8 (3)	C12—C13—H13A	109.00
O11—C11—C1	123.4 (3)	C12—C13—H13B	109.00
O12—C11—C1	110.8 (2)	C12—C13—H13C	109.00
O12—C12—C13	112.5 (3)	H13A—C13—H13B	109.00
C4—C41—C42	120.7 (2)	H13A—C13—H13C	110.00
C4—C41—C46	121.1 (2)	H13B—C13—H13C	110.00
C42—C41—C46	118.1 (2)	C41—C42—H42	119.00
C41—C42—C43	121.2 (2)	C43—C42—H42	119.00
C42—C43—C44	119.1 (2)	C42—C43—H43	120.00
Br4—C44—C43	119.30 (18)	C44—C43—H43	121.00
Br4—C44—C45	119.5 (2)	C44—C45—H45	120.00
C43—C44—C45	121.2 (2)	C46—C45—H45	120.00
C44—C45—C46	119.8 (3)	C41—C46—H46	120.00
C41—C46—C45	120.6 (2)	C45—C46—H46	120.00
C6—C61—C62	122.6 (2)	C61—C62—H62	120.00
C6—C61—C66	119.5 (2)	C63—C62—H62	120.00
C62—C61—C66	117.7 (3)	C62—C63—H63	120.00
C61—C62—C63	120.9 (3)	C64—C63—H63	120.00
C62—C63—C64	120.7 (3)	C63—C64—H64	120.00
C63—C64—C65	119.6 (4)	C65—C64—H64	120.00
C64—C65—C66	120.5 (4)	C64—C65—H65	120.00
C61—C66—C65	120.6 (3)	C66—C65—H65	120.00
C2—C1—H1	108.00	C61—C66—H66	120.00
C6—C1—H1	108.00	C65—C66—H66	120.00
C11—C1—H1	108.00		
			
C12—O12—C11—O11	−2.1 (4)	C4—C5—C6—C1	−48.4 (2)
C12—O12—C11—C1	175.5 (2)	C4—C5—C6—C61	−172.20 (18)
C11—O12—C12—C13	78.0 (4)	C1—C6—C61—C62	−77.1 (3)
C6—C1—C2—O2	154.3 (3)	C1—C6—C61—C66	97.7 (3)
C6—C1—C2—C3	−30.8 (3)	C5—C6—C61—C62	47.1 (3)
C11—C1—C2—O2	30.2 (4)	C5—C6—C61—C66	−138.2 (3)
C11—C1—C2—C3	−154.9 (2)	C4—C41—C42—C43	−180.0 (2)
C2—C1—C6—C5	53.5 (3)	C46—C41—C42—C43	0.1 (3)
C2—C1—C6—C61	179.9 (2)	C4—C41—C46—C45	−179.1 (3)
C11—C1—C6—C5	177.74 (18)	C42—C41—C46—C45	0.9 (4)
C11—C1—C6—C61	−55.9 (2)	C41—C42—C43—C44	−1.1 (3)
C2—C1—C11—O11	−121.9 (3)	C42—C43—C44—Br4	−179.29 (17)
C2—C1—C11—O12	60.5 (3)	C42—C43—C44—C45	1.3 (4)
C6—C1—C11—O11	113.2 (3)	Br4—C44—C45—C46	−179.7 (2)
C6—C1—C11—O12	−64.5 (3)	C43—C44—C45—C46	−0.3 (4)
O2—C2—C3—C4	176.6 (3)	C44—C45—C46—C41	−0.8 (4)
C1—C2—C3—C4	1.6 (4)	C6—C61—C62—C63	174.7 (3)
C2—C3—C4—C5	3.8 (4)	C66—C61—C62—C63	−0.1 (4)
C2—C3—C4—C41	−173.9 (2)	C6—C61—C66—C65	−174.6 (3)
C3—C4—C5—C6	20.8 (3)	C62—C61—C66—C65	0.4 (5)
C41—C4—C5—C6	−161.42 (19)	C61—C62—C63—C64	−0.2 (5)
C3—C4—C41—C42	−29.7 (3)	C62—C63—C64—C65	0.3 (6)
C3—C4—C41—C46	150.3 (2)	C63—C64—C65—C66	0.0 (6)
C5—C4—C41—C42	152.5 (2)	C64—C65—C66—C61	−0.3 (6)
C5—C4—C41—C46	−27.5 (3)		

Symmetry codes: (i) −*x*, −*y*+1, −*z*; (ii) *x*, −*y*+1/2, *z*−1/2; (iii) *x*−1, *y*, *z*−1; (iv) *x*−1, −*y*+1/2, *z*−1/2; (v) −*x*, −*y*, −*z*; (vi) −*x*, *y*−1/2, −*z*+1/2; (vii) *x*, −*y*+1/2, *z*+1/2; (viii) −*x*, *y*+1/2, −*z*+1/2; (ix) −*x*+1, −*y*+1, −*z*+1; (x) −*x*+1, −*y*, −*z*+1; (xi) *x*+1, −*y*+1/2, *z*+1/2; (xii) *x*+1, *y*, *z*+1.

### Hydrogen-bond geometry (Å, °)

**Table:**

Cg is the centroid of the C41–C46 ring.

**Table:**

*D*—H···*A*	*D*—H	H···*A*	*D*···*A*	*D*—H···*A*
C42—H42···O2^v^	0.93	2.58	3.276 (3)	132
C45—H45···O11^viii^	0.93	2.54	3.288 (4)	138
C1—H1···Cg^vii^	0.98	2.77	3.648 (2)	150

Symmetry codes: (v) −*x*, −*y*, −*z*; (viii) −*x*, *y*+1/2, −*z*+1/2; (vii) *x*, −*y*+1/2, *z*+1/2.
